# Do Gene Variants Influencing Adult Adiposity Affect Birth Weight? A Population-Based Study of 24 Loci in 4,744 Danish Individuals

**DOI:** 10.1371/journal.pone.0014190

**Published:** 2010-12-01

**Authors:** Ehm A. Andersson, Kasper Pilgaard, Charlotta Pisinger, Marie N. Harder, Niels Grarup, Kristine Færch, Camilla Sandholt, Pernille Poulsen, Daniel R. Witte, Torben Jørgensen, Allan Vaag, Oluf Pedersen, Torben Hansen

**Affiliations:** 1 Hagedorn Research Institute, Gentofte, Denmark; 2 Steno Diabetes Center, Gentofte, Denmark; 3 Research Centre for Prevention and Health, Glostrup University Hospital, Glostrup, Denmark; 4 Faculty of Health Sciences, University of Copenhagen, Copenhagen, Denmark; 5 Faculty of Health Sciences, University of Southern Denmark, Odense, Denmark; Innsbruck Medical University, Austria

## Abstract

**Background:**

Several obesity risk alleles affecting adult adiposity have been identified by the recent wave of genome wide association studies. We aimed to examine the potential effect of these variants on fetal body composition by investigating the variants in relation to birth weight and ponderal index of the newborn.

**Methodology/Principal Findings:**

Midwife records from the Danish State Archives provided information on mother's age, parity, as well as birth weight, birth length and prematurity of the newborn in 4,744 individuals of the population-based Inter99 study. Twenty-four risk alleles showing genome-wide associations with adult BMI and/or waist circumference were genotyped. None of the 24 risk variants tested showed an association with birth weight or ponderal index after correction for multiple testing. Birth weight was divided into three categories low (≤10^th^ percentile), normal (10^th^–90^th^ percentile) and high birth weight (≥90th percentile) to allow for non-linear associations. There was no difference in the number of risk alleles between the groups (p = 0.57). No interactions between each risk allele and birth weight in the prediction of adult BMI were observed. An obesity risk score was created by summing up risk alleles. The risk score did not associate with fetal body composition. Moreover there was no interaction between the risk score and birth weight/ponderal index in the prediction of adult BMI.

**Conclusion:**

24 common variants associated with adult adiposity did not affect or interact with birth weight among Danes suggesting that the effects of these variants predominantly arise in the post-natal life.

## Introduction

Obesity has become a worldwide health epidemic with increasing prevalence. Several studies have demonstrated that birth weight is associated with Body Mass index (BMI) in later life [1]. Most studies report a positive linear relationship between birth weight and adult BMI [1], while others have suggested a U-shaped relationship, where both low and high birth weight predispose to an increased adult BMI [2], [3].

Although the mechanisms behind the association are far from clarified, both genetic and environmental factors seem to play a role. From an environmental point of view the association between high birth weight and adult obesity may in part be explained by alterations in the intrauterine environment mediated by maternal obesity or maternal hyperglycaemia during pregnancy, as it has been shown that newborns of diabetic or overweight mothers are heavier [4]. It has been suggested that these maternal factors might alter the programming of hypothalamic pathways involved in appetite regulation and energy expenditure [5].

Few studies have investigated the genetic contribution of the association between birth weight and adult obesity. A total of 24 gene variants, influencing adult adiposity, have been identified by the recent wave of genome-wide association studies (GWAS) [6], [7], [8], [9], [10], [11], [12], [13], [14], [15]. Only variants in or near *TMEM18*, *ETV5* (*SFRS10*), *BDNF*, *FTO* and *MC4R* loci have been examined for associations with birth weight previously. No association with birth weight has been observed for variants in or near *TMEM18*, *ETV5* (*SFRS10*), *BDNF* and *MC4R* loci [7], [16], [17], [18], [19], [20]. While the majority of studies have found no association between variants in the *FTO* locus and birth weight [6], [18], [21], [22], [23], one study of 4,693 individuals found an *FTO* obesity risk allele to be associated with higher BMI and ponderal index at birth [20].

Moreover, an obesity-risk-allele score, comprising variants in or near the *FTO, MC4R, TMEM18, GNPDA2, KCTD15, NEGR1, BDNF* and *ETV5 (SFRS10*) loci has been tested for an association with birth weight in 7,146 individuals [17] and a modest association with BMI at birth was observed.

These previous studies do not indicate that adult obesity loci may contribute strongly to the variation in birth weight, however, the effect of many obesity risk variants recently identified have not yet been investigated.

We hypothesized that some of the genetic variants associated with adult adiposity may have an impact on fetal growth and therefore explain part of the link between birth weight and subsequent risk of obesity. Therefore, we aimed to test the single effect of each variant as well as the combined effect of the 24 variants on birth weight and ponderal index in the Inter99 population. Since previous studies have reported both high and low birth weight to associate with BMI, it is possible that these phenotypes and the genetic risk variants may have an interactive effect on adult BMI, e.g. the risk variants may have a stronger effect in those born with birth weights lying near the ends of the distribution. For that reason, we also aimed to test for an interactive effect between high/low birth weight and the genetic variants in the prediction of adult BMI.

## Methods

### Study population

Ethics statement: All participants gave written informed consent and the protocol was in accordance with the Helsinki Declaration, approved by Copenhagen County ethic committee and registered with ClinicalTrials.gov (NCT00289237). Individuals examined in the present study were from the Danish Inter99 Study, which at baseline comprised 6,784 individuals living in the suburb of Copenhagen. The Inter99 is a population-based randomised non-pharmacological intervention study aimed to prevent ischemic heart disease. The study was conducted at the Research Centre for Prevention and Health in Glostrup, Denmark (www.inter99.dk) [24]. For 4,744 participants, midwife journals were traced through the Danish State Archives. These journals contained information on mother's age and parity as well as birth weight, birth length and maturity status of the newborn [25]. Ponderal index was calculated as birth weight (kg)/birth length (m^3^). Information about the mother's diabetes status was obtained by a questionnaire during the baseline visit in 1999–2001. The age of onset of maternal diabetes was not registered.

Pregnancies were considered at term when gestation attained 36 completed weeks and did not exceed 41 completed weeks. Participants born preterm (n = 446) or as part of a multiple pregnancy (n = 85) were excluded, since these newborns have a lower birth weight presumably due to non-genetic factors. Preterm birth was a clinical assessment by the midwife based on the pregnancy due date together with clinical signs of prematurity. The final number of individuals included in the study was 4,213. All individuals were Danes by self report.

### SNP selection

In order to include variants affecting overall adiposity, SNPs were selected based on their previous genome-wide association with adult BMI/obesity and waist circumference. BMI and waist circumference are highly correlated measures of adiposity and although variants in or near *MC4R*, *NRXN3*, *TFAP2B* and *MSRA* were initially identified to associate with waist circumference [12], [13], [15] they most likely affect overall adiposity and was therefore included in the present study. Another SNP, rs17782313, in the *MC4R* locus in strong linkage disequilibrium with rs12970134 (HapMap CEU: r2 = 0.81) was simultaneously found to associate strongly with BMI and risk of obesity [7]. *NRXN3* rs10146997 was also associated with BMI and the association with waist circumference was completely attenuated when adjusting for BMI [13]. *TFAP2B* rs987237 was at genome-wide significance level associated with BMI in addition to waist circumference in the main paper [12]. *MSRA* rs545854 was only weakly associated with BMI but the signal for waist circumference was abolished when adjusting for BMI [12] suggesting that this SNP also most likely affects overall adiposity.

### Genotyping

Genotyping was performed as described previously for 19 of the variants [26]. The remaining variants were genotyped using KASPar® genotyping (KBioscience, Hoddesdon, UK). All genotyping success rates were >95% with an error rate of <1% estimated in >470 replicate samples. All genotypes obeyed Hardy–Weinberg equilibrium in the Danish population (p>0.01).

### Statistical analysis

All statistical analyses were performed using RGui version 2.8.1 (available at http://www.r-project.org). Z-scores were calculated as z-score  =  (x-mean)/sd. The effect of the genetic variants on birth weight and ponderal index was calculated using linear regression models adjusted for sex, maternal diabetes (yes vs. no/NA) and parity (0, 1, 2, 3 or ≥4). No transformation of data was performed. Only additive genetic models were considered assuming a constant change in birth weight per risk allele and p<0.05 was considered statistically significant. The Bonferroni corrected threshold for 24 tests was p<0.0021. Individuals were classified into groups of birth weight; newborns born at or below the sex-specific 10^th^ percentile in the Inter99 population were defined as having low birth weight, whereas those born at or above the 90^th^ percentile were defined as having high birth weight. Age and sex-adjusted interaction analyses between each risk allele and low-normal-high birth weight were performed in relation to adult BMI. An un-weighted genetic risk score was calculated by summing up risk alleles assuming similar effects of all alleles. A total of 2,926 individuals had a genotype call for all variants and could be included in the analyses. The association between the risk score and adult BMI was performed by using linear regression adjusted for age and sex. The association between the risk score and birth weight/ponderal index was tested by linear regression adjusted for sex, maternal diabetes status and parity. The association between the risk score and groups of birth weight was calculated by a one-way anova. The interaction between the risk score and birth weight were tested in relation to adult BMI adjusted for age and sex using linear regression. Statistical power was estimated using 1000 simulations. We used the empirical variance of the observed traits adjusted for sex, maternal diabetes and parity to simulate phenotypes from a normal distribution, so that variance across genotypes is drawn from the estimated variance.

## Results

**Figure 1:**
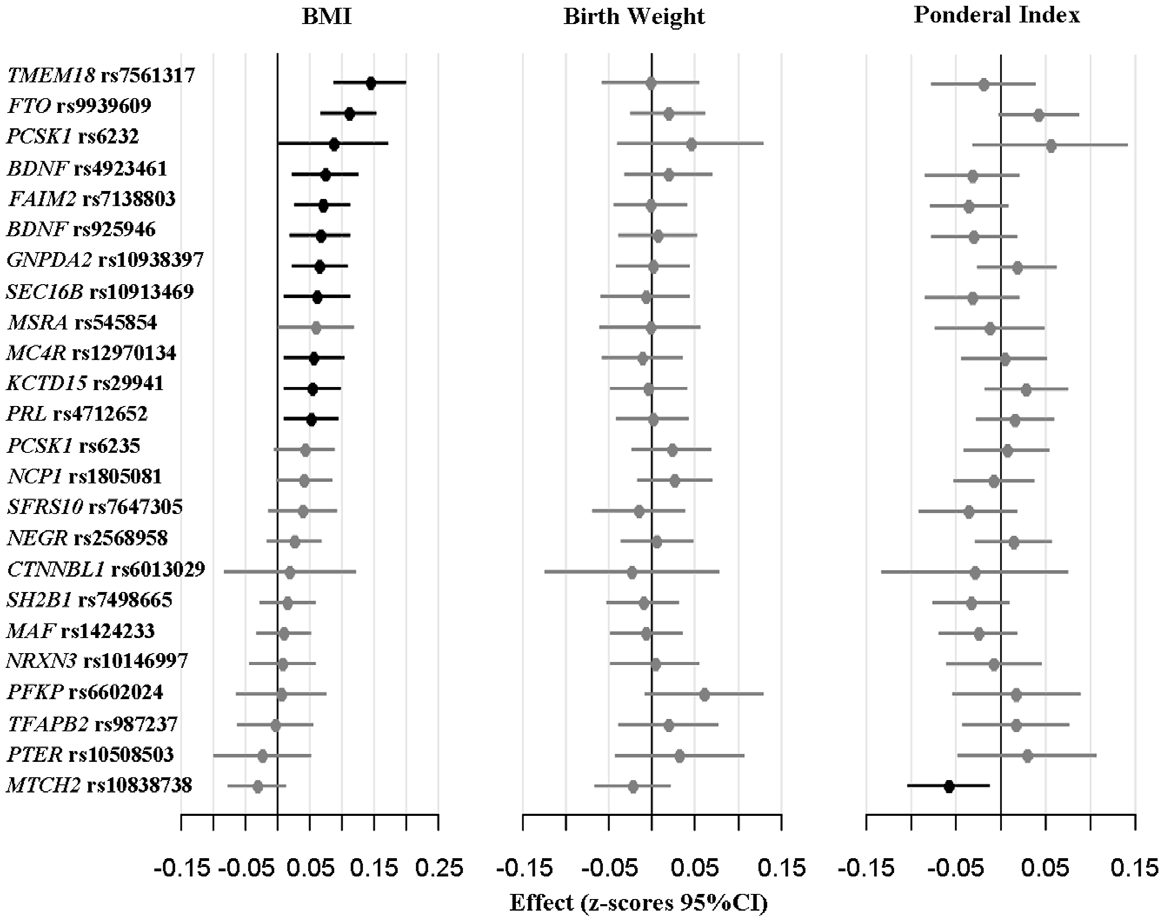
Individual associations between obesity risk variants and BMI, birth weight and ponderal index in the subsample of Inter99 with birth weight data available (n = 4,213). Effect sizes and 95%CI are giving as z-scores. The variants are ordered according to their effect on adult BMI and their effects are given for the allele previously reported to increase adult adiposity. Black dots and lines indicate statistically significant associations (*p*<0.05), whereas grey indicate non-significant. General linear models assuming additive genetic models are applied for all variants. Analyses of BMI are adjusted for age and sex. Analyses of birth weight and ponderal index are adjusted for sex, maternal diabetes status and parity. CI; confidence interval.

After exclusion of premature and multiple births, 4,213 individuals with birth weight data were available for the present analyses in Inter99. In this subsample 11 of 24 gene variants were significantly associated with adult BMI, whereas 21 variants had estimated effect sizes in the direction previously reported (Figure 1).

The 24 genetic variants were then investigated for an association with birth weight and ponderal index in the Inter99 population (Figure 1, supplementary table S1–S2). None of the 24 risk variants showed an association with birth weight and only the *MTCH2*-rs10838738 obesity risk-allele was nominally associated with reduced ponderal index (β = −0.13 kg/m^3^, 95%CI: [−0.23; −0.03], p = 0.01).

Birth weight was divided into three categories low (≤10^th^ percentile), normal (10^th^–90^th^ percentile) and high birth weight (≥90th percentile). Each risk variant was tested for an interaction with these groups of birth weight in the prediction of adult BMI. No interactions were observed between each risk allele and groups of birth weight in the prediction of adult BMI (Supplementary Table S1).

**Figure 2:**
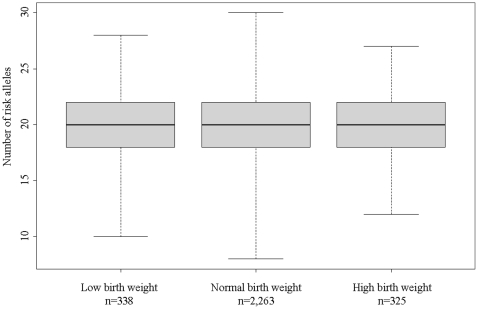
Boxplot showing the number of obesity risk alleles in individuals with low, normal and high birth weight, respectively (n = 2,926). The black line is the median number of risk alleles, the grey boxes represent the interquartile range and the whiskers denote the range of risk alleles within each group. Individuals with low birth weight (n = 338) were born at or below the sex-specific 10^th^ percentile of birth weight in the Inter99 population, while individuals with high birth weight (n = 325) were born at or above the sex-specific 90^th^ percentile of birth weight.

Although there were no individual SNPs related to birth weight, we assessed the potential summed effect of the risk alleles by constructing an un-weighted obesity risk allele score. The risk allele score was strongly associated with adult BMI in this subsample of Inter99 with birth weight data and genotype data available for all 24 variants (n = 2,926) (per allele: zscore  = 0.04 95%CI [0.03;0.06], p = 1.2*10^−13^), (Table 1). The risk score was however not associated with quantitative birth weight or ponderal index (Table 1). Moreover, there was no difference in the number of risk alleles between those with low, normal or high birth weight (p = 0.57) (Figure 2). We also tested the extremes of the risk score by comparing the 10% carrying most risk alleles with the 10% carrying least risk alleles. Although there was a large difference in adult BMI between these two extremes, no associations with birth weight and ponderal index were observed (Table 1). Moreover, the risk score and extremes of the risk score did not interact with quantitative birth weight or birth weight groups in order to predict adult BMI (Table 1).

**Table 1:** Analyses of obesity risk scores and adult BMI, birth weight and ponderal index in a subsample of Inter99 with available genotype and birth weight data (n = 2,926).

Obesity risk score (full spectrum)	Adult BMI	Birth Weight	Ponderal Index
Effect per allele: z-score (95%CI)	0.04 (0.03;0.06)	0.002 (−0.01;0.01)	−0.007 (−0.02;0.005)
*P*	1.2*10^−13^	0.76	0.26
*P_int_*	-	0.34	0.27
*P_int2_*	-	0.069	-

Obesity risk score (lower 10% vs. upper 10%)	Adult BMI	Birth Weight	Ponderal Index
Effect per allele: z-score (95%CI)	0.5 (0.4;0.7)	0.002 (−0.13;0.14)	−0.1 kg/m^3^ (−2.6;0.04)
*P*	3.4*10^−12^	0.98	0.16
*P_int_*	-	0.93	0.40
*P_int2_*	-	0.19	-

Effect sizes and 95%CI are giving as z-scores. The obesity risk score (full spectrum) and extremes of the risk score (lower 10% vs. upper 10%) was tested for associations with adult BMI, birth weight and ponderal index. *P_int_* denotes the *p*-value for the interactions between the risk scores and quantitative birth weight and quantitative ponderal index in the prediction of adult BMI. *P_int2_* denotes the *p*-value for the interactions between the risk score and groups of birth weight (low-medium-high) in the prediction of adult BMI. CI; confidence interval.

## Discussion

We found that none of the 24 genotypes which have a confirmed association to adult adiposity are associated with birth weight. The *MTCH2*-rs10838738 obesity risk variant was associated with a decreased ponderal index, however this association did not remain significant after accounting for multiple testing and it is therefore likely to be a chance finding. Moreover, an obesity risk score was strongly associated with adult BMI in the subsample of Inter99 with birth weight data available, but it did not associate with birth weight and ponderal index. We did not observe any interactions between risk variants and birth weight in the prediction of adult BMI.

Our results therefore suggest that the risk variants affecting adult adiposity do not have a strong impact on size at birth and therefore are not likely to explain the association between birth weight and adult BMI. Supporting this notion, previous studies have not found altered birth weight in carriers of mutations causing monogenic forms of obesity [27]. The results of the present study are therefore in line with the majority of previous studies which have failed to show any associations between obesity risk variants and birth weight [6], [18], [21], [22], [23]. However one recent well-powered study by Elks *et al.* found a modest association between an obesity risk score comprising alleles from *FTO, MC4R, TMEM18, GNPDA2, KCTD15, NEGR1, BDNF* and *ETV5 (SFRS10*) loci and BMI at birth in 7,146 individuals. When constructing a similar risk score in 3,496 individuals from Inter99 with genotype and birth weight data available no significant association was found (data not shown). This may be due to a lower statistical power in the present study, but as discussed by Elks and colleagues the obesity risk variants seem to have a larger effect on weight gain during infancy and childhood.

It is well known that the central nervous system and especially areas of hypothalamus play key roles in whole body energy regulation. The majority of the genes, in which mutations cause monogenic obesity, are involved in the control of appetite and food intake through the hypothalamus [28]. Similarly, most of the 24 loci analysed in the present study are suggested to include genes with hypothalamic expression, although the specific molecular mechanisms behind these links are not well characterised [28].

We hypothesized that the examined gene variants known to affect adult adiposity could be affecting the individual from as early as pre-natal life by facilitating energy uptake and storage and thus weight regulation of the fetus. In the present study we did, however, not find any genetic support for this hypothesis, suggesting that the genetic components of fetal and adult weight gain are largely independent. During pregnancy, multiple factors including nutrient supply, placental function and available space are determinants of fetal growth, indicating a major importance of external factors on the growth of the fetus. Thus, it is likely that the central appetite regulation is mainly activated or only matures in post natal life, when the external environment becomes a less marked limiting factor for the intake of nutrients and subsequent growth. Moreover, the lack of an interactive effect between genetic risk and birth weight suggests that these variables influence adult BMI and risk of obesity independently in this cohort.

We have recently reported that specific type 2 diabetes risk alleles influence birth weight in the Inter99 population [29] and a large genome-wide association study on birth weight has recently reported two variants to associate with birth weight, one of which is a known type 2 diabetes locus [21]. No obesity loci were identified by this powerful approach. The mechanism by which the type 2 diabetes loci influence birth weight is presumably by influencing fetal insulin availability. Although 8 of the 24 obesity gene variants investigated in the present study influence adult insulin secretion in the Inter99 population (unpublished data), they do not associate with birth weight, suggesting that their effect on insulin secretion may be predominantly observed later in life, presumably as a result of increased insulin resistance in carriers of the obesity risk alleles.

A total of 11 of the 24 investigated genes were significantly associated with adult BMI in the subsample of Inter99 with birth weight data available. For these variants, the effects of the risk alleles are therefore most likely to arise during post-natal life. The statistical power to detect minor changes in quantitative variables such as birth weight may be low for the remaining variants. In the Danish Inter99 study, we have more than 80% statistical power to detect effects in birth weight of 30 g, 35 g and 45 g assuming minor allele frequencies of 10%, 20% and 40%, respectively, and we can thus exclude effects on birth weight within this range.

It could have been of interest if the alleles with the strongest effect on adult BMI also had the strongest effect on birth weight although not reaching statistical significance in this study sample. We have illustrated this in figure 1 by plotting the z-scores of BMI, birth weight and ponderal index. However, by visual comparison of the effect on adult BMI with the effect on birth weight and ponderal index from the figure, no tendency in this direction seems to exist.

A recent study comprising 20 of the variants investigated in the present study, found that the 20 variants explained only 4.5% of the obesity status in the Inter99 cohort [26]. Therefore hypothetical, not yet identified risk variants affecting pathways important for both fetal and adult weight gain may still exist.

Lack of exact information regarding gestational age between week 37 and 42 is a limitation of this study. However, none of the investigated variants have to our knowledge been associated with gestational age and thus an even genotype distribution can be anticipated. Moreover, in a recent meta-analysis three studies without information of gestational age did not introduce heterogeneity in to the results [21].

It is known from previous studies that the maternal genotype can interact with the fetal genotype in the determination of birth weight suggesting a complex pattern for the regulation of fetal growth [30]. We do not have information on parental genotypes at present but it could be of major interest to test if maternal obesity risk genotypes may affect fetal growth and thereby explain part of the association between high birth weight and subsequent risk of obesity.

In conclusion, 24 common variants associated with adult adiposity did not have a strong effect on birth weight in the Danish Inter99 population. Also no interactions between the genetic variants and birth weight in the prediction of adult BMI was observed.

## Supporting Information

Table S1 Fetal genotype and birth weight in 4,213 individuals from the Danish Inter99 study population. Bonferroni threshold for 24 test is p<0.0021. Data are means +/− standard deviation of birth weight (g). Effects and p-values are calculated assuming an additive genetic model adjusted for sex, maternal diabetes status and parity. Pint, p-value for the interaction between each variant and groups of birth weight in relation to adult BMI. CI, confidence interval.
(0.15 MB DOC)

Table S2 Fetal genotype and ponderal index in 4,213 individuals from the Danish Inter99 study population. Bonferroni threshold for 24 test is p<0.0021. Data are means +/− standard deviation of ponderal index (kg/m3). Effects and p-values are calculated assuming an additive genetic model adjusted for sex, maternal diabetes status and parity. CI, confidence interval.
(0.08 MB DOC)
