# Supplementary material for: Do Gene Variants Influencing Adult Adiposity Affect Birth Weight? A Population-Based Study of 24 Loci in 4,744 Danish Individuals
Source: PLoS One. 2010 Dec 1;5(12):e14190. doi: 10.1371/journal.pone.0014190 (PMC2995733; doi:10.1371/journal.pone.0014190)
Supplement: Table S1 — Fetal genotype and birth weight in 4,213 individuals from the Danish Inter99 study population. Bonferroni threshold for 24 test is p<0.0021. Data are means +/− standard deviation of birth weight (g). Effects and p-values are calculated assuming an additive genetic model adjusted for sex, maternal diabetes status and parity. Pint, p-value for the interaction between each variant and groups of birth weight in relation to adult BMI. CI, confidence interval. (0.15 MB DOC) [file pone.0014190.s001.doc]

Table 1: Fetal genotype and birth weight in 4,213 individuals from the Inter99 study

| **SNP** | **Risk allele frequency** | **Mean birth weight (g)** | | | **Effect**  **(95% CI)**  **per allele** | ***P*** | ***P_int_*** |
| --- | --- | --- | --- | --- | --- | --- | --- |
|  |  | Genotype (number of obesity risk alleles) | | |  |  |  |
|  |  | 0 | 1 | 2 |  |  |  |
| *FTO* rs9939609 | 0.41 | 3,490±438 | 3,481±455 | 3510±449 | 8  (-11, 28) | 0.41 | 0.69 |
| *TMEM18* rs7561317 | 0.83 | 3,487±501 | 3,492±444 | 3,488±450 | -1  (-26, 25) | 0.96 | 0.09 |
| *PCSK1* rs6232 | 0.07 | 3,485±449 | 3,499±444 | 3,600±467 | 20  (-18, 58) | 0.30 | 0.48 |
| *PCSK1* rs6235 | 0.29 | 3,482±444 | 3,490±459 | 3,515±435 | 10  (-11, 31) | 0.35 | 0.16 |
| *CTNNBL1* rs6013029 | 0.05 | 3,489±450 | 3,488±441 | 3,138±243 | -11  (-56, 35) | 0.65 | 0.26 |
| *SH2B1* rs7498665 | 0.41 | 3,486±457 | 3,496±446 | 3,474±445 | -5  (-24, 14) | 0.62 | 0.22 |
| *KCTD15* rs29941 | 0.68 | 3,484±471 | 3,491±451 | 3,490±446 | -2  (-22, 18) | 0.85 | 0.87 |
| *MTCH2* rs10838738 | 0.35 | 3,490±443 | 3,492±457 | 3,458±447 | -10  (-31, 10) | 0.32 | 0.35 |
| *GNPDA2* rs10938397 | 0.41 | 3,482±437 | 3,496±460 | 3,477±439 | 0  (-19, 20) | 0.97 | 0.55 |
| *PFKP* rs6602024 | 0.10 | 3,482±446 | 3,517±458 | 3,426±429 | 27  (-4, 58) | 0.09 | 0.96 |
| *SFRS10* rs7647305 | 0.80 | 3,540±444 | 3,485±442 | 3,488±455 | -7  (-31, 17) | 0.57 | 0.33 |
| *NCP1* rs1805081 | 0.57 | 3,474±446 | 3,484±445 | 3,500±458 | 12  (-8, 31) | 0.24 | 0.81 |
| *MAF* rs1424233 | 0.47 | 3,497±454 | 3,484±448 | 3,490±446 | 3  (-22, 16) | 0.75 | 0.69 |
| *PTER* rs10508503 | 0.91 | 3,340±516 | 3,489±462 | 3,490±448 | 14  (-49, 20) | 0.41 | 0.83 |
| *PRL* rs4712625 | 0.41 | 3,485±452 | 3,499±445 | 3,473±464 | 0  (-19, 19) | 0.99 | 0.87 |
| *FAIM2* rs7138803 | 0.40 | 3,485±444 | 3,504±451 | 3,459±460 | -1  (-20, 18) | 0.92 | 0.18 |
| *BDNF* rs4923461 | 0.78 | 3,510±423 | 3,479±438 | 3,494±459 | 9  (-15, 32) | 0.46 | 0.94 |
| *BDNF* rs925946 | 0.31 | 3,489±449 | 3,488±444 | 3,513±458 | 3  (-18, 24) | 0.80 | 0.53 |
| *NEGR.* rs2568958 | 0.59 | 3,474±450 | 3,498±448 | 3,487±448 | 2  (-17, 21) | 0.81 | 0.59 |
| *SEC16B* rs10913469 | 0.21 | 3,492±450 | 3,487±453 | 3,489±419 | -4  (-27, 20) | 0.76 | 0.59 |
| *MC4R* rs12970134 | 0.28 | 3,495±452 | 3,480±452 | 3,490±429 | -5  (-26, 16) | 0.63 | 0.33 |
| *NRXN3* rs10146997 | 0.21 | 3,485±443 | 3,496±461 | 3,476±439 | 1  (-22, 25) | 0.9 | 0.44 |
| *TFAPB2* rs987237 | 0.17 | 3,484±448 | 3,495±453 | 3,482±441 | 8  (-18, 35) | 0.54 | 0.46 |
| *MSRA* rs545854 | 0.15 | 3,490±453 | 3,481±431 | 3,522±498 | -1  (-28, 25) | 0.93 | 0.09 |

Bonferroni threshold for 24 test is p<0.0021. Data are means ± standard deviation of birth weight (g). Effects and p-values are calculated assuming an additive genetic model adjusted for sex, maternal diabetes status and parity. *P_int_*, p-value for the interaction between each variant and groups of birth weight in relation to adult BMI. CI, confidence interval.

Supplementary Table 1: Fetal genotype and ponderal index in 4,213 individuals from the Danish Inter99 study population

| **SNP** | **Risk allele frequency** | **Mean ponderal index (kg/m^3^)** | | | **Effect**  **(95% CI)**  **per allele** | ***P*** |
| --- | --- | --- | --- | --- | --- | --- |
|  |  | Genotype (number of obesity risk alleles) | | |  |  |
|  |  | 0 | 1 | 2 |  |  |
| *FTO*  rs9939609 | 0.41 | 24.7±2.3 | 24.7±2.2 | 25.0±2.2 | 0.10  (-0.01, 0.20) | 0.07 |
| *TMEM18*  rs7561317 | 0.83 | 25.0±2.2 | 24.8±2.3 | 24.8±2.3 | -0.08  (-0.18, 0.09) | 0.52 |
| *PCSK1*  rs6232 | 0.07 | 24.7±2.3 | 24.8±2.1 | 25.5±2.1 | 0.13  (-0.07, 0.32) | 0.21 |
| *PCSK1*  rs6235 | 0.29 | 24.8±2.2 | 24.8±2.3 | 24.8±2.3 | 0.02  (-0.09, 0.12) | 0.78 |
| *CTNNBL1*  rs6013029 | 0.05 | 24.8±2.3 | 24.7±2.2 | 23.7±1.9 | -0.07  (-0.30, 0.17) | 0.58 |
| *SH2B1*  rs7498665 | 0.41 | 24.8±2.3 | 24.8±2.2 | 24.7±2.3 | -0.08  (-0.17, 0.02) | 0.13 |
| *KCTD15*  rs29941 | 0.68 | 24.7±2.3 | 24.7±2.2 | 24.8±2.3 | 0.06  (-0.04, 0.17) | 0.23 |
| *MTCH2*  rs10838738 | 0.35 | 24.8±2.3 | 24.8±2.3 | 24.5±2.3 | -0.13  (-0.23, -0.03) | 0.01 |
| *GNPDA2*  rs10938397 | 0.41 | 24.7±2.3 | 24.8±2.3 | 24.7±2.2 | 0.04  (-0.06, 0.14) | 0.42 |
| *PFKP*  rs6602024 | 0.10 | 24.8±2.3 | 24.8±2.3 | 24.5±2.4 | 0.04  (-0.12, 0.20) | 0.64 |
| *SFRS10*  rs7647305 | 0.80 | 24.8±2.2 | 24.8±2.2 | 24.7±2.3 | -0.08  (-0.21, 0.04) | 0.19 |
| *NCP1*  rs1805081 | 0.57 | 24.8±2.3 | 24.8±2.3 | 24.7±2.3 | -0.02  (-0.12, 0.08) | 0.74 |
| *MAF*  s1424233 | 0.47 | 24.8±2.3 | 24.7±2.3 | 24.7±2.2 | -0.06  (-0.17, 0.04) | 0.26 |
| *PTER*  rs10508503 | 0.91 | 24.8±2.3 | 24.8±2.3 | 23.9±2.1 | -0.06  (-0.24, 0.11) | 0.47 |
| *PRL*  rs4712625 | 0.41 | 24.7±2.3 | 24.9±2.3 | 24.7±2.2 | 0.03  (-0.06, 0.13) | 0.47 |
| *FAIM2*  rs7138803 | 0.40 | 24.8±2.3 | 24.8±2.3 | 24.6±2.4 | -0.08  (-0.18, 0.02) | 0.11 |
| *BDNF*  rs4923461 | 0.78 | 24.9±2.2 | 24.8±2.3 | 24.7±2.2 | -0.07  (-0.19, 0.05) | 0.25 |
| *BDNF*  rs925946 | 0.31 | 24.8±2.3 | 24.7±2.2 | 24.8±2.4 | -0.07  (-0.18, 0.05) | 0.21 |
| *NEGR1*  rs2568958 | 0.59 | 24.7±2.3 | 24.8±2.2 | 24.8±2.3 | 0.03  (-0.07, 0.13) | 0.53 |
| *SEC16B*  rs10913469 | 0.21 | 24.8±2.3 | 24.7±2.3 | 24.8±2.1 | -0.07  (-0.19, 0.05) | 0.24 |
| *MC4R*  rs12970134 | 0.28 | 24.8±2.3 | 24.8±2.2 | 24.8±2.5 | 0.01  (-0.10, 0.12) | 0.87 |
| *NRXN3*  rs10146997 | 0.21 | 24.8±2.3 | 24.7±2.3 | 24.8±2.4 | -0.02  (-0.14, 0.10) | 0.78 |
| *TFAPB2*  rs987237 | 0.17 | 24.7±2.3 | 24.8±2.3 | 24.6±2.1 | 0.04  (-0.10, 0.17) | 0.59 |
| *MSRA*  rs545854 | 0.15 | 24.8±2.3 | 24.7±2.2 | 24.8±2.5 | -0.03  (-0.17, 0.11) | 0.68 |

Bonferroni threshold for 24 test is p<0.0021. Data are means ± standard deviation of ponderal index (kg/m^3^). Effects and p-values are calculated assuming an additive genetic model adjusted for sex, maternal diabetes status and parity. CI, confidence interval.
