# Supplementary material for: Do Gene Variants Influencing Adult Adiposity Affect Birth Weight? A Population-Based Study of 24 Loci in 4,744 Danish Individuals
Source: PLoS One. 2010 Dec 1;5(12):e14190. doi: 10.1371/journal.pone.0014190 (PMC2995733; doi:10.1371/journal.pone.0014190)
Supplement: Table S2 — Fetal genotype and ponderal index in 4,213 individuals from the Danish Inter99 study population. Bonferroni threshold for 24 test is p<0.0021. Data are means +/− standard deviation of ponderal index (kg/m3). Effects and p-values are calculated assuming an additive genetic model adjusted for sex, maternal diabetes status and parity. CI, confidence interval. (0.08 MB DOC) [file pone.0014190.s002.doc]

Table S2: Fetal genotype and ponderal index in 4,213 individuals from the Danish Inter99 study population

| **SNP** | **Risk allele frequency** | **Mean ponderal index (kg/m^3^)** | | | **Effect**  **(95% CI)**  **per allele** | ***P*** |
| --- | --- | --- | --- | --- | --- | --- |
|  |  | Genotype (number of obesity risk alleles) | | |  |  |
|  |  | 0 | 1 | 2 |  |  |
| *FTO*  rs9939609 | 0.41 | 24.7±2.3 | 24.7±2.2 | 25.0±2.2 | 0.10  (-0.01, 0.20) | 0.07 |
| *TMEM18*  rs7561317 | 0.83 | 25.0±2.2 | 24.8±2.3 | 24.8±2.3 | -0.08  (-0.18, 0.09) | 0.52 |
| *PCSK1*  rs6232 | 0.07 | 24.7±2.3 | 24.8±2.1 | 25.5±2.1 | 0.13  (-0.07, 0.32) | 0.21 |
| *PCSK1*  rs6235 | 0.29 | 24.8±2.2 | 24.8±2.3 | 24.8±2.3 | 0.02  (-0.09, 0.12) | 0.78 |
| *CTNNBL1*  rs6013029 | 0.05 | 24.8±2.3 | 24.7±2.2 | 23.7±1.9 | -0.07  (-0.30, 0.17) | 0.58 |
| *SH2B1*  rs7498665 | 0.41 | 24.8±2.3 | 24.8±2.2 | 24.7±2.3 | -0.08  (-0.17, 0.02) | 0.13 |
| *KCTD15*  rs29941 | 0.68 | 24.7±2.3 | 24.7±2.2 | 24.8±2.3 | 0.06  (-0.04, 0.17) | 0.23 |
| *MTCH2*  rs10838738 | 0.35 | 24.8±2.3 | 24.8±2.3 | 24.5±2.3 | -0.13  (-0.23, -0.03) | 0.01 |
| *GNPDA2*  rs10938397 | 0.41 | 24.7±2.3 | 24.8±2.3 | 24.7±2.2 | 0.04  (-0.06, 0.14) | 0.42 |
| *PFKP*  rs6602024 | 0.10 | 24.8±2.3 | 24.8±2.3 | 24.5±2.4 | 0.04  (-0.12, 0.20) | 0.64 |
| *SFRS10*  rs7647305 | 0.80 | 24.8±2.2 | 24.8±2.2 | 24.7±2.3 | -0.08  (-0.21, 0.04) | 0.19 |
| *NCP1*  rs1805081 | 0.57 | 24.8±2.3 | 24.8±2.3 | 24.7±2.3 | -0.02  (-0.12, 0.08) | 0.74 |
| *MAF*  s1424233 | 0.47 | 24.8±2.3 | 24.7±2.3 | 24.7±2.2 | -0.06  (-0.17, 0.04) | 0.26 |
| *PTER*  rs10508503 | 0.91 | 24.8±2.3 | 24.8±2.3 | 23.9±2.1 | -0.06  (-0.24, 0.11) | 0.47 |
| *PRL*  rs4712625 | 0.41 | 24.7±2.3 | 24.9±2.3 | 24.7±2.2 | 0.03  (-0.06, 0.13) | 0.47 |
| *FAIM2*  rs7138803 | 0.40 | 24.8±2.3 | 24.8±2.3 | 24.6±2.4 | -0.08  (-0.18, 0.02) | 0.11 |
| *BDNF*  rs4923461 | 0.78 | 24.9±2.2 | 24.8±2.3 | 24.7±2.2 | -0.07  (-0.19, 0.05) | 0.25 |
| *BDNF*  rs925946 | 0.31 | 24.8±2.3 | 24.7±2.2 | 24.8±2.4 | -0.07  (-0.18, 0.05) | 0.21 |
| *NEGR1*  rs2568958 | 0.59 | 24.7±2.3 | 24.8±2.2 | 24.8±2.3 | 0.03  (-0.07, 0.13) | 0.53 |
| *SEC16B*  rs10913469 | 0.21 | 24.8±2.3 | 24.7±2.3 | 24.8±2.1 | -0.07  (-0.19, 0.05) | 0.24 |
| *MC4R*  rs12970134 | 0.28 | 24.8±2.3 | 24.8±2.2 | 24.8±2.5 | 0.01  (-0.10, 0.12) | 0.87 |
| *NRXN3*  rs10146997 | 0.21 | 24.8±2.3 | 24.7±2.3 | 24.8±2.4 | -0.02  (-0.14, 0.10) | 0.78 |
| *TFAPB2*  rs987237 | 0.17 | 24.7±2.3 | 24.8±2.3 | 24.6±2.1 | 0.04  (-0.10, 0.17) | 0.59 |
| *MSRA*  rs545854 | 0.15 | 24.8±2.3 | 24.7±2.2 | 24.8±2.5 | -0.03  (-0.17, 0.11) | 0.68 |

Bonferroni threshold for 24 test is p<0.0021. Data are means ± standard deviation of ponderal index (kg/m^3^). Effects and p-values are calculated assuming an additive genetic model adjusted for sex, maternal diabetes status and parity. CI, confidence interval.
